# Retraction Note: Electrochemical and hot corrosion behaviour of annealed AlCoCrFeNi HEA coating over steel

**DOI:** 10.1038/s41598-025-14601-z

**Published:** 2025-08-12

**Authors:** N. Radhika, Niveditha Noble, Adeolu Adesoji Adediran

**Affiliations:** 1 Department of Mechanical Engineering, Amrita School of Engineering, Amrita Vishwa Vidyapeetham, Coimbatore, India; 2 Department of Mechanical Engineering, Landmark University, P.M.B. 1001, Omu-Aran, Kwara State Nigeria; 3 Department of Mechanical Engineering Science, University of Johannesburg, Auckland Park Kingsway, Johannesburg, South Africa

Retraction of: *Scientific Reports* 10.1038/s41598-024-55962-1, published online 07 March 2024

The Editors have retracted this Article.

After publication, concerns were raised regarding several SEM images presented in this Article, specifically Figures 15e, 16e, 17e, and 18b. The concerns were brought to the Editors’ attention after a third party raised concerns about the authenticity of the data and potential unauthorized use of materials.

- Figures 17e and 18b were replaced during a post-publication correction^1^, but the newly submitted images lacked metadata and appeared different in structure and surface morphology compared to the published figures.
- For Figures 15e and 16e, the authors were unable to provide the original data upon request. These figures also show strong similarities to images shared by a third party, although the SEM specifications differ from those reported in the Article.

The Editors therefore no longer have confidence in the research presented in this work.

N. Radhika and Adeolu Adesoji Adediran disagree with this retraction. Niveditha Noble did not respond to the correspondence from Editors about the retraction.
